# The association between zinc and prostate cancer development: A systematic review and meta-analysis

**DOI:** 10.1371/journal.pone.0299398

**Published:** 2024-03-20

**Authors:** Shahrzad Shahrokhi Nejad, Zahra Golzari, Moein Zangiabadian, Amir Abbas Salehi Amniyeh Khozani, Rasoul Ebrahimi, Seyed Aria Nejadghaderi, Azadeh Aletaha

**Affiliations:** 1 School of Medicine, Shahid Beheshti University of Medical Sciences, Tehran, Iran; 2 Endocrinology and Metabolism Research Center, Institute of Basic and Clinical Physiology Sciences, Kerman University of Medical Sciences, Kerman, Iran; 3 Systematic Review and Meta-analysis Expert Group (SRMEG), Universal Scientific Education and Research Network (USERN), Tehran, Iran; 4 Evidence Based Medicine Research Center, Endocrinology and Metabolism Clinical Sciences Institute, Tehran University of Medical Sciences, Tehran, Iran; 5 Endocrinology and Metabolism Research Center, Endocrinology and Metabolism Clinical Sciences Institute, Tehran University of Medical Sciences, Tehran, Iran; University of Ha’il, SAUDI ARABIA

## Abstract

**Background:**

Prostate cancer is affecting males globally, with several complications. Zinc can play roles in cancers. We aimed to clarify the association between zinc levels or intake with prostate cancer development.

**Methods:**

We searched PubMed, EMBASE, Cochrane Central Register of Controlled Trials (CENTRAL), and Web of Science until May 1, 2023. We included case-controls and cross-sectionals that measured zinc level and/or intake in patients with prostate cancer or cohorts that evaluated the association between zinc and prostate cancer development. Studies that did not have a healthy control group were excluded. Joanna Briggs Institute was used for quality assessment. Publication bias was evaluated using Egger’s and Begg’s tests and funnel plot.

**Results:**

Overall, 52 studies (n = 44 case controls, n = 4 cohorts, and n = 4 cross sectionals) with a total number of 163909 participants were included. Serum (standardized mean difference (SMD): -1.11; 95% confidence interval (CI): -1.67, -0.56), hair (SMD: -1.31; 95% CI: -2.19, -0.44), and prostatic fluid or tissue zinc levels (SMD: -3.70; 95% CI: -4.90, -2.49) were significantly lower in prostate cancer patients. There were no significant differences in nail zinc level and zinc intake between those with prostate cancer and healthy controls. There was no publication bias except for serum and hair zinc levels based on Begg’s and Egger’s tests, respectively. The mean risk of bias scores were 4.61 in case-controls, eight in cohorts, and seven in cross-sectionals.

**Conclusions:**

Overall, high zinc levels might have a protective role in prostate cancer, which can be used as a therapeutic or preventive intervention. Future large-scale studies are needed to confirm the association.

## Introduction

Prostate cancer was the second cancer with the highest incidence among males in 2019 globally [1]. In 2020, approximately 1.41 million individuals were newly diagnosed with prostate cancer and it led to more than 375 thousand deaths worldwide [1]. Moreover, complications like urinary tract symptoms have a significant impact on the physical and mental well-being of patients [2–4]. Genetic and environmental factors play roles in prostate cancer development so the disparities in prostate cancer incidence worldwide suggest that dietary factors may contribute to these variations. However, the specific components of the diet that contribute to this phenomenon have not been identified [5, 6].

Zinc has been identified as a dietary factor that may play a protective role in prostate cancer [7]. The levels of zinc are tightly controlled as it is involved in many physiological processes [8]. Zinc accumulates in the prostate ten times higher than any other tissue and this accumulation plays a vital role in maintaining the overall health of the prostate gland [9, 10]. Epidemiological studies have shown that there is a notable decrease in serum zinc levels in different types of cancer, such as head and neck, breast, gastrointestinal tract, female genital tract, gallbladder, lung, and thyroid cancers [11–13].

While experimental data has supported the beneficial impact of zinc in prostate cancer, various epidemiological studies have yielded conflicting results [14, 15]. Some studies have found that zinc intake reduces the likelihood of developing prostate cancer and its mortality rate [16–18]. Conversely, other studies have linked high zinc consumption to advanced prostate cancer [19]. However, several observations have indicated that dietary or supplemental zinc intake is not associated with the risk of prostate cancer or its progression [20–22]. Previous meta-analysis studies have been conducted on this topic [23–25]. However, considering that their search dates back to years ago and more recent studies with larger sample sizes have been conducted [26, 27], we would like to update them and also consider their limitations in the current study. In this regard, a case-control study and meta-analysis was conducted in 2016, whereas it did not perform quality assessment [24]. Also, its search date went back to 2016 and only included 17 studies [24]. In another meta-analysis which searched the literature up to 2015, the association between serum zinc and different prostate diseases were evaluated, although it was not focused on prostate cancer [28]. The meta-analyses by Stratton et al. and Gumulec et al. also evaluated the broad range of different types of supplemental vitamins and minerals or assessed zinc effects on different types of epithelial malignancies [23, 25]. Overall, the previous studies are out-of-dated or did not evaluate the effects of zinc concentrations in different samples on prostate cancer. So, our study considered zinc levels in various specimens, such as blood serum, prostate tissue, nail, and scalp hair, as well as zinc supplementation and dietary intake. Zinc level in each one is profoundly measured and correlated to the risk of prostate cancer in various studies, but none of the studies have gathered all in a systematic review. Herein, we aimed to conduct a systematic review and meta-analysis to evaluate the association between zinc levels or intake and prostate cancer development.

## Materials and methods

This systematic review and meta-analysis was in accordance with the Preferred Reporting Items for Systematic Reviews and Meta-Analyses (PRISMA) guidelines [29]. The protocol was registered in the International Prospective Register of Systematic Reviews (PROSPERO) with registration ID CRD42023439347.

### Search strategy and study selection

We searched PubMed, EMBASE, Cochrane Central Register of Controlled Trials (CENTRAL), and Web of Science up to May 1, 2023. We used the following terms: ("zinc" OR "zinc compounds" OR "Zn" OR "zinc citrate" OR "zinc sulfate") AND ("prostatic neoplasm*" OR "prostate cancer" OR "prostate malignancy") (S1 Table). No filters on any search field, such as language and study types were implemented. Backward and forward citation searching were performed. Also, the first 300 results of the Google Scholar search engine were evaluated as the grey literature search [30].

The inclusion criteria consisted of case-controls, cohorts, and analytical cross-sectionals on patients with prostate cancer of any stage which evaluated the effects of zinc supplementation or zinc levels on prostate cancer. The outcome of interest was the occurrence of prostate cancer. The studies should have reported or provided calculable odds ratio (OR), hazard ratio (HR), or relative risk (RR) with a 95% confidence interval (CI).

The exclusion criteria were studies that included patients with types of cancers other than prostate cancer, studies reporting levels of vitamins/minerals other than zinc, and studies reporting outcomes other than prostate cancer occurrence (e.g., mortality and survival). Also, in vitro and in vivo studies, animal studies, case reports, case series, editorials, commentaries, letters, review articles, notes, news, book chapters, meta-analyses, and re-analyses of previously published articles were excluded. The records were imported and deduplicated using the EndNote software (Clarivate Analytics). The records were divided into two groups, and two different pairs of reviewers (AASAK & RE–ZG & SSN) screened each one independently by title and abstract search in the first step. Then, the full texts of the studies from the previous screening were evaluated. Any disagreements were resolved by discussing and consulting with the lead investigator (SAN).

### Data extraction

A form in Microsoft Office Excel was used for data extraction. Two pairs of independent researchers (AASAK & RE–ZG & SSN) extracted the following information from each study: first author name; year of publication; study design; country where the research was conducted; study population; definition of case or exposure groups and controls; age range and mean age in cases and controls; follow-up duration; source of sampling; comorbidities; smoking, alcohol consumption, and other risk factors; prostate cancer ascertainment; and methods of zinc measurement. Any discrepancies were resolved by discussing or consulting with a third author.

### Quality assessment

The included studies were divided into two groups and two different pairs of reviewers (AASAK & RE–ZG & SSN) assessed the quality of each one independently using the Joanna Briggs Institute (JBI), critical appraisal tools for case controls, cohorts, and analytical cross-sectionals [31]. A third reviewer (SAN) was consulted if there were any discrepancies.

### Statistical analysis

The pooled ORs with 95% CIs for dichotomous data and the pooled standardized mean difference (SMD) with 95% CIs for continuous data were assessed using random or fixed-effects models. The random-effects model was used because of the estimated methodological heterogeneity of the true effect sizes. The between-study heterogeneity was assessed by Cochran’s Q and the I-square statistic. I-square values of more than 50% were considered high heterogeneity [32]. Stratified analysis was done for SMD calculation according to the source of zinc sampling. This method was conducted to consider the effects of zinc level in different types of samples or zinc intake. The median and interquartile range were converted to mean and standard deviation for SMD calculation [33]. Publication bias was evaluated using Egger’s and Begg’s tests as well as the funnel plot (p<0.05 was considered indicative of statistically significant publication bias as well as funnel plot asymmetry) [34]. If the results of Egger’s and Begg’s tests were incoherent, the trim-and-fill method was used to find probable missing studies [35, 36]. The funnel plot was not used for publication bias assessment in analysis with fewer than ten studies [37].

## Results

**Fig 1:**
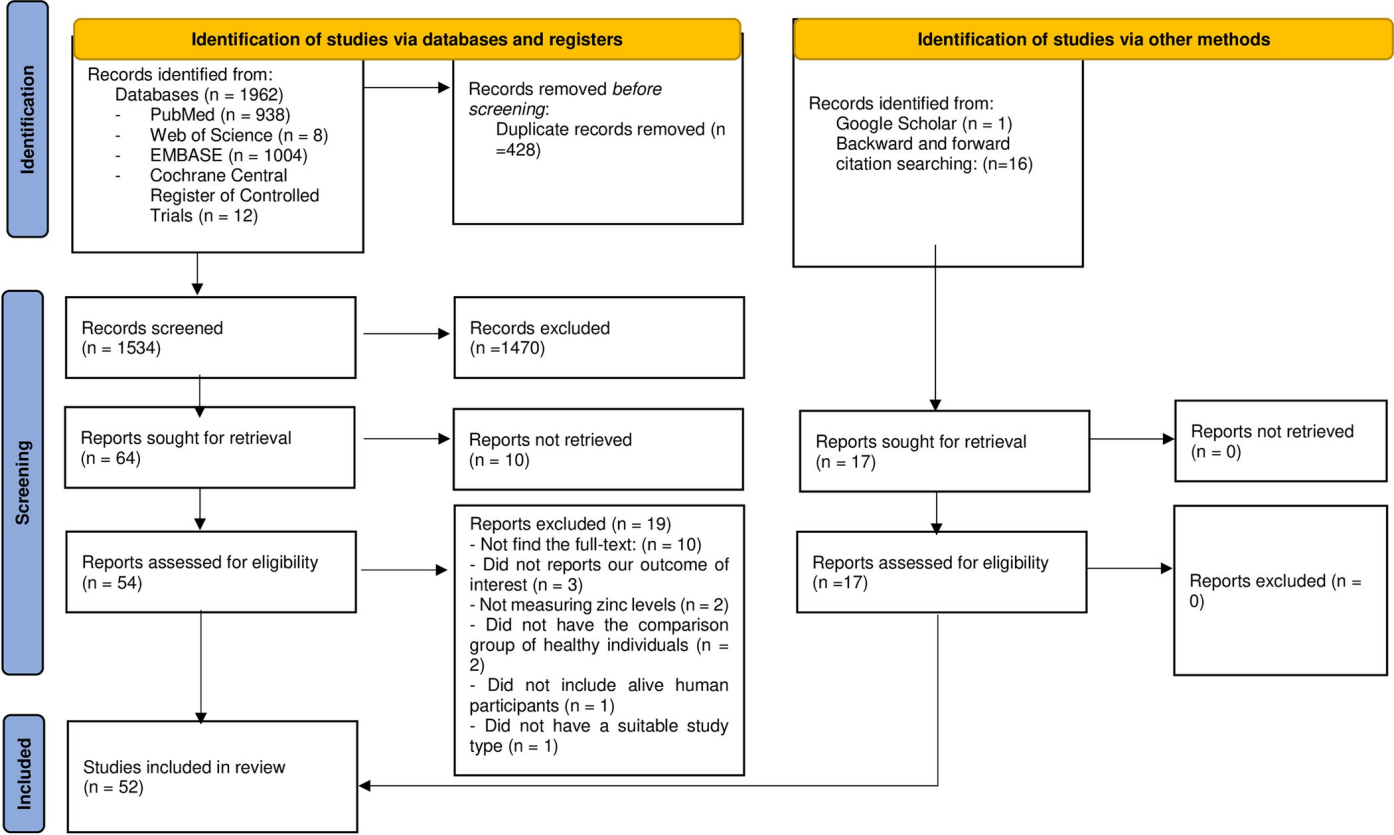
Study selection process.

A total of 1962 hits were found through online database searching. After removing duplicated results, 1524 studies were evaluated in the title and abstract screening, and 1470 records were excluded in this step. Then, 54 studies were evaluated in full-text reviewing. We could not find the full text of ten studies [38–47], three did not report our outcomes of interest [48–50], two were excluded due to not measuring zinc levels [51, 52], two did not have the comparison group of healthy individuals [53, 54], one did not include alive human participants [55], and one did not have a suitable study type [56]. Overall, 35 studies were included in the full-text review [19, 21, 22, 26, 57–87]. Moreover, 16 studies were found through backward [20, 24, 88–98] and forward citation searching [99–101], and one study from searching Google Scholar [102]. Finally, 52 articles were included in this systematic review, of which forty-four were case-controls [19, 21, 22, 24, 57–65, 67, 69–73, 75–82, 84–92, 94, 95, 97–102], four were cohorts [20, 26, 66, 74], and four were analytical cross-sectional studies [68, 83, 93, 96]. Overall, 50 studies were included in quantitative synthesis and two in qualitative synthesis (Fig 1).

### Study characteristics

Twelve studies were conducted in the United States [19–21, 24, 26, 66, 72–75, 80, 84], nine in Nigeria [57, 59, 68, 76–78, 83, 88, 94], five in Turkey [58, 70, 79, 85, 89], four in China [91–93, 96], three in India [61, 65, 69], two in Saudi Arabia [82, 95], two in Russia [86, 87], two in Germany [62, 63], one study in each other countries, such as Poland [60], Venezuela [90], Spain [67], Sudan [99], Singapore [102], Iraq [100], Serbia [97], United Kingdom [98], Iran [101], Malaysia [71], Italy [64], Sweden [22], and Pakistan [81]. The follow-up duration of cohort studies ranged from seven to about 28 years [20, 26, 66, 74]. In 36 studies, prostate cancer was confirmed by a histopathological examination [19, 20, 22, 24, 26, 57–59, 61–64, 67–69, 71–74, 76–78, 80, 82–89, 92, 97, 100–102]; in two studies it was confirmed by prostate-specific antigen (PSA) test [81, 94], and in one study it was confirmed by clinical data and current international clinical staging method [93]. In other 13 studies, prostate cancer diagnosis method was not described [21, 60, 65, 66, 70, 75, 79, 90, 91, 95, 96, 98, 99] (Table 1 and S2 Table).

**Table 1:** Study characteristics.

First Author	Publication year	Country	Study design	Total participants	Total mean (SD) age	Zinc measurement method	Source of sampling
Yiwen Zhang [26]	2022	United States	Cohort	47240	66.14 (0.27)	NA	FFQ
Mehmet Kaba [70]	2014	Turkey	Case-control	62	64.06 (1.31)	AAS	Serum
KS Adedapo [88]	2012	Nigeria	Case-control	120	66.85 (1.66)	AAS using a direct method	Serum
Collins Amadi [57]	2020	Nigeria	Case-control	440	69.35 (0.35)	AAS using a direct method	Serum
Katarzyna Białkowska [60]	2018	Poland	Case-control	394	77	ICP-MS technique	Serum
Ayşe Eken [89]	2016	Turkey	Case-control	131	61.27 (7.14)	AAS with a Zeaman Background Correction, flame atomic absorption spectrometer	Serum
Yenny Gómez [90]	2007	Venezuela	Case-control	40	54.29 (NA)	ETA-AAS	Prostatic fluid
Jingkang Guo [91]	2007	NA	Case-control	115	NA	ICP-MS	Scalp hair
Enrique Gutiérrez-González [67]	2018	Spain	Case-control	1961	65.95 (0.25)	Dietary zinc intake estimated	FFQ
Martin Igbokwe [68]	2021	Nigeria	Cross-sectional	82	71.78 (1.05)	PIXE	Toe-nail
Lina Mustafa khedir Abdelmajid [99]	2022	Sudan	Case-control	60	NA	AAS	Serum
Alan R. Kristal [20]	2010	United States	Cohort	9559	62.77(0.38)	FFQ and a structured supplement-use questionnaire	FFQ
Marion M. Lee [92]	1998	China	Case-control	398	NA	FFQ and face-to-face interviews	FFQ
Xiao-Meng Li [93]	2005	China	Cross-sectional	3940	68.47 (1.05)	Deproteinization method using a Perkin-Elmer 503 AAS	Serum
Jue Tao Lim [102]	2019	Singapore	Case-control	255	NA	ICP-MS	Serum
Abeer M. Mahmoud [24]	2016	United States	Case-control	208	66.30 (0.24)	Block FFQ	FFQ
Rana Kareem Mohammed [100]	2015	Iraq	Case-control	50	NA	AAS	Serum
Augusta Chinyere Nsonwu-Anyanwu [76]	2022	Nigeria	Case-control	90	36.76 (2.04)	Wet acid digestion method	Serum
Wasiu Eniola Olooto [78]	2021	Nigeria	Case-control	75	NA	AAS	Serum
Bridget Obiageli Onyema-iloh [94]	2014	Nigeria	Case-control	100	NA	AAS	Serum
Saleh A. K. Saleh [95]	2017	Saudi Arabia	Case-control	174	69.10 (1.70)	ICP-MS	Scalp hair
Chao Tan [96]	2011	China	Cross-sectional	113	NA	ICP-MS	Scalp hair
H.D. Vlajinac [97]	1997	Serbia	Case-control	303	71.16 (NA)	Measurements of consumption using standard cups, spoons, and slices	Zinc intake
Victor C. Wakwe [83]	2019	Nigeria	Cross-sectional	440	69.35 (0.38)	AAS	Serum
Elizabeth G. Willden [98]	1975	United Kingdom	Case-control	92	NA	AAS	Serum
Hasan Yari [101]	2015	Iran	Case-control	72	65.30 (1.20)	Polarography	Serum
Vladimir Zaichick [86]	2019	Russia	Case-control	146	61.32 (2.94)	EDXRF	Prostatic fluid
Michael F. Leitzmann [74]	2003	United States	Cohort	46974	54.34 (0.64)	Zinc intake	FFQ
J.O.Ogunlewe [77]	1989	Nigeria	Case-control	127	63.81 (2.34)	AAS	Serum and prostatic tissue
T.Goel [65]	2006	India	Case-control	80	NA	AAS	Serum
Ahmet Aydin [58]	2006	Turkey	Case-control	85	65.44 (1.37)	Flame AAS	Serum
V.YE.Zaichick [87]	1996	Russia	Case-control	91	56.18 (7.90)	XRF	Prostatic fluid
D.W.West [84]	1991	United States	Case-control	1037	NA	FFQ	FFQ
Song-Yi Park [21]	2013	United States	Case-control	1175	68.97 (0.09)	ICP-MS	Serum
Alejandro Gonzalez [66]	2009	United States	Cohort	35242	NA	Zinc intake	FFQ
Laurence N. Kolonel [72]	1988	United States	Case-control	1351	NA	FFQ	FFQ
A. Feustel [62]	1986	Germany	Case-control	147	53.70 (NA)	Flame AAS	Serum and erythrocytes
Golgis Karimi [71]	2012	Malaysia	Case-control	100	72.05 (0.35)	ICP-MS	Hair and nail
Silvano Gallus[64]	2007	Italy	Case-control	2745	62.20 (NA)	Zinc intake	FFQ
Elizabeth A. Platz [80]	2002	United States	Case-control	342	66.03 (0.05)	Furnace AAS and flame AAS	Nail
M. Jain [69]	1994	India	Case-control	50	NA	AAS	Serum and prostatic tissues
Alan R. Kristal [73]	1999	United States	Case-control	1363	NA	Zinc intake	FFQ
M. I. Yilmaz [85]	2004	Turkey	Case-control	121	65.01	Flame AAS	Serum
Habibe Ozmen [79]	2006	Turkey	Case-control	41	69.32 (3.10)	AAS	Serum
Swen-Olof Andersson [22]	1996	Sweden	Case-control	1056	70.65 (0.05)	Zinc intake	FFQ
Muhammad Abdul Qayyum [81]	2014	Pakistan	Case-control	394	51.70 (NA)	AAS	Serum, scalp hair, and nail
Pamela Christudoss [61]	2011	India	Case-control	83	NA	AAS	Serum and tissue
Saleh A.K. Saleh [82]	2020	Saudi Arabia	Case-control	92	67.17 (1.04)	ICP-MS	Serum
Onyinyechi Bede-Ojimadu [59]	2023	Nigeria	Case-control	273	NA	ICP-MS	Urine and serum
Lois D. Mcbean [75]	1974	United States	Case-control	95	49.00 (NA)	AAS	Serum
A.Feustel [63]	1989	Germany	Case-control	75	68.75 (NA)	Flame AAS	Serum
Yuqing Zhang [19]	2009	United States	Case-control	4110	60.15 (1.48)	Supplementation use using a structured questionnaire	FFQ

Abbreviations: NA: not available; AAS: atomic absorption spectrometry; FFQ: food frequency questionnaire; ICP-MS: inductively coupled plasma mass spectroscopy; PIXE: Particle induced X-ray emission; EDXRF: energy dispersive X-ray fluorescent; XRF: X-ray fluorescence.

The total number of participants was 163909 which ranged from 40 to 47240 participants in individual studies with mean age ranging from 36.76 to 77 years. Atomic absorption spectrometry (AAS) was the most common method for zinc measurement. The sources of sampling were different which included serum [21, 57–63, 65, 69, 70, 75–79, 81–83, 85, 88, 89, 93, 94, 98–102], erythrocyte [62], prostatic fluid or tissue [61, 69, 77, 86, 87, 90], hair [71, 81, 91, 95, 96], and nail [68, 71, 80, 81]. Also, eleven studies used a questionnaire to assess levels of zinc intake [19, 20, 24, 26, 64, 66, 67, 73, 74, 84, 92]. Out of these 11 studies, eight studies grouped patients based on a zinc level threshold [19, 20, 26, 64, 66, 73, 74, 84], hence using odds ratio to report results; and four studies compared zinc intake levels using a mean intake measurement [24, 67, 74, 92] (Table 1 and S2 Table).

### Meta-analysis and publication bias

#### Serum zinc level

**Fig 2:**
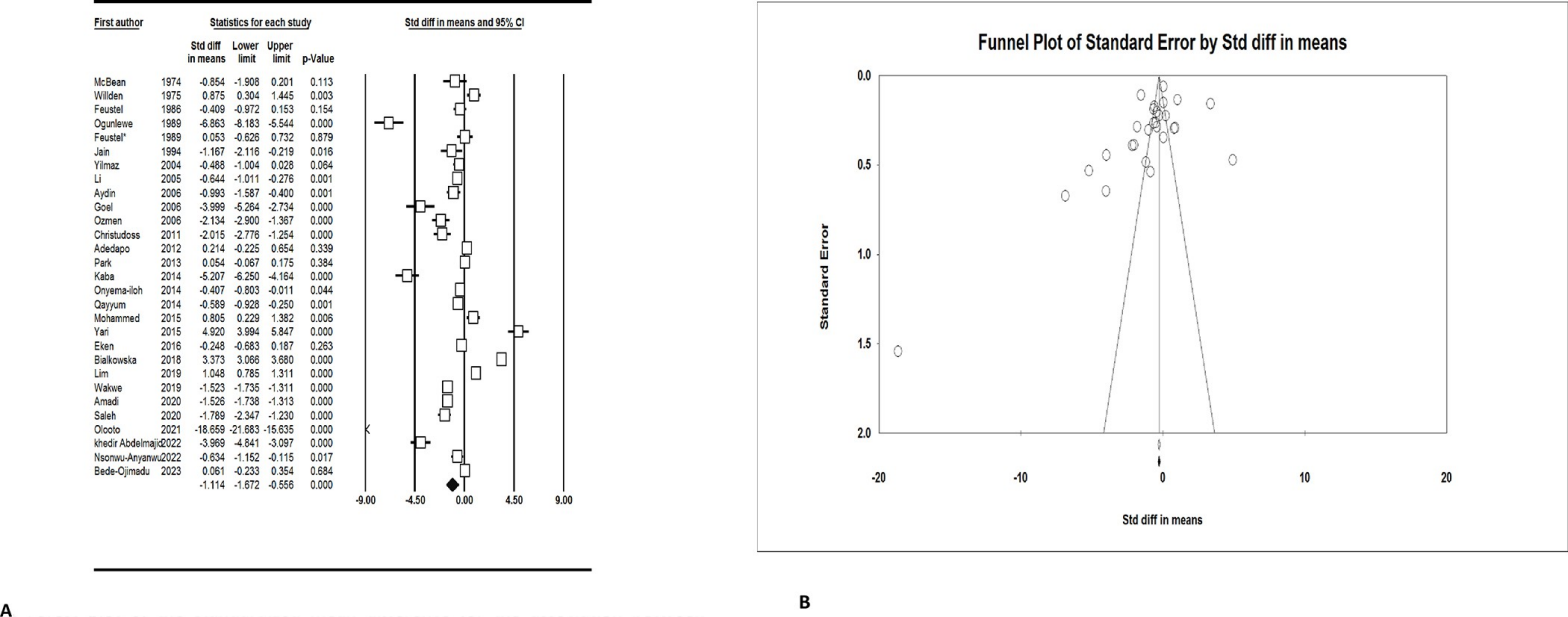
A. Forest plot of the standardized mean difference for the association between prostate cancer and serum zinc level. B. Funnel plot for serum zinc level and prostate cancer.

Serum zinc level was significantly lower in prostate cancer patients (SMD: -1.11; 95% CI: -1.67, -0.56) (Fig 2A). There was a significant publication bias according to the Begg’s test (p = 0.03) and funnel plot, however, no significant publication bias was found in the Egger’s test (p = 0.15) (Fig 2B).

#### Zinc intake

**Fig 3:**
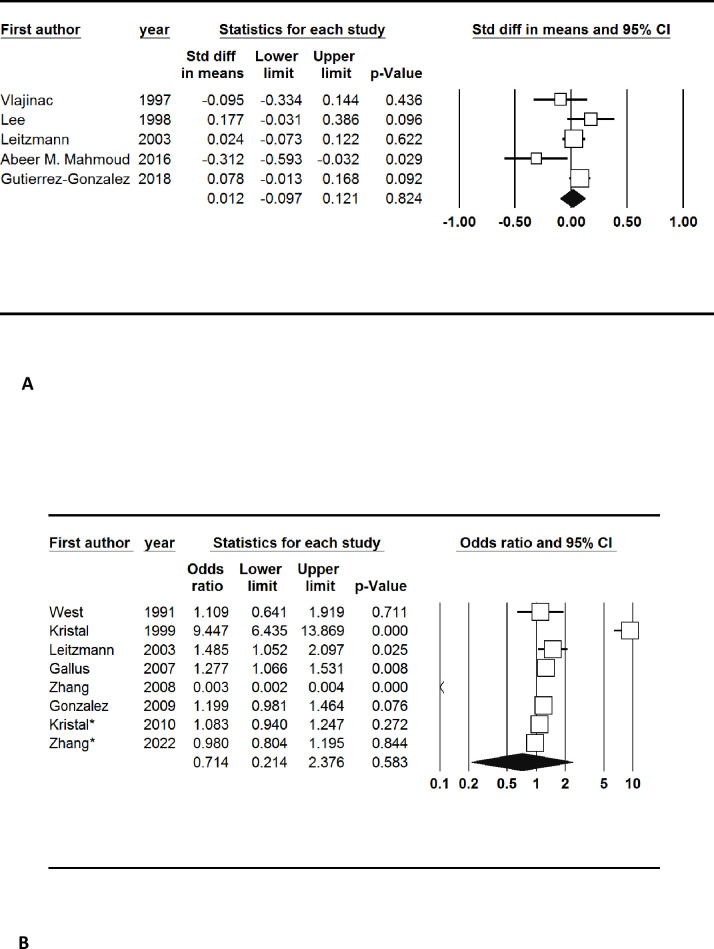
A. Forest plot of the standardized mean difference for the association between prostate cancer and zinc intake. B. Forest plot of the odds ratio for the association between prostate cancer and zinc intake.

Five studies reported the mean levels of zinc intake per day. The pooled results showed that zinc intake was non-significantly higher in prostate cancer patients (SMD: 0.01, 95% CI: -0.10, 0.12). There was also no publication bias for the measurement of serum zinc intake (Begg’s test: 0.22 and Egger’s test: 0.35) (Fig 3A).

Eight studies categorized the participants based on the levels of zinc intake and reported the number of participants in each category. There was no significant association between zinc intake and prostate cancer (OR: 0.71; 95% Cl: 0.21, 2.38). Also, there was no publication bias (Begg’s test: 0.54 and Egger’s test: 0.68) (Fig 3B).

#### Nail zinc level

**Fig 4:**
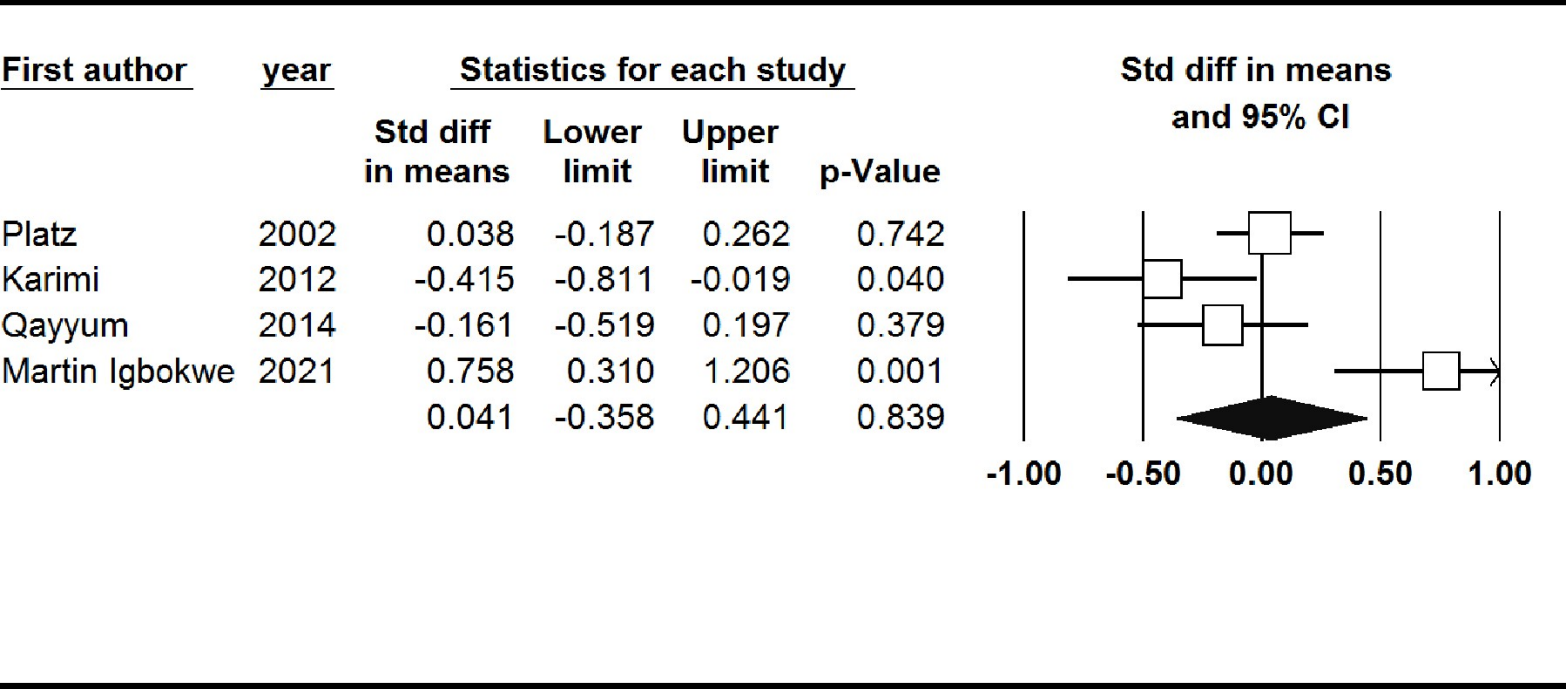
Forest plot of the standardized mean difference for the association between prostate cancer and nail zinc level.

Nail zinc level was non-significantly higher in patients with prostate cancer (SMD: 0.04, 95% CI: -0.36, 0.44). There was also not a significant publication bias (Begg’s test: 1.00, Egger’s test: 0.85) (Fig 4).

#### Hair zinc level

**Fig 5:**
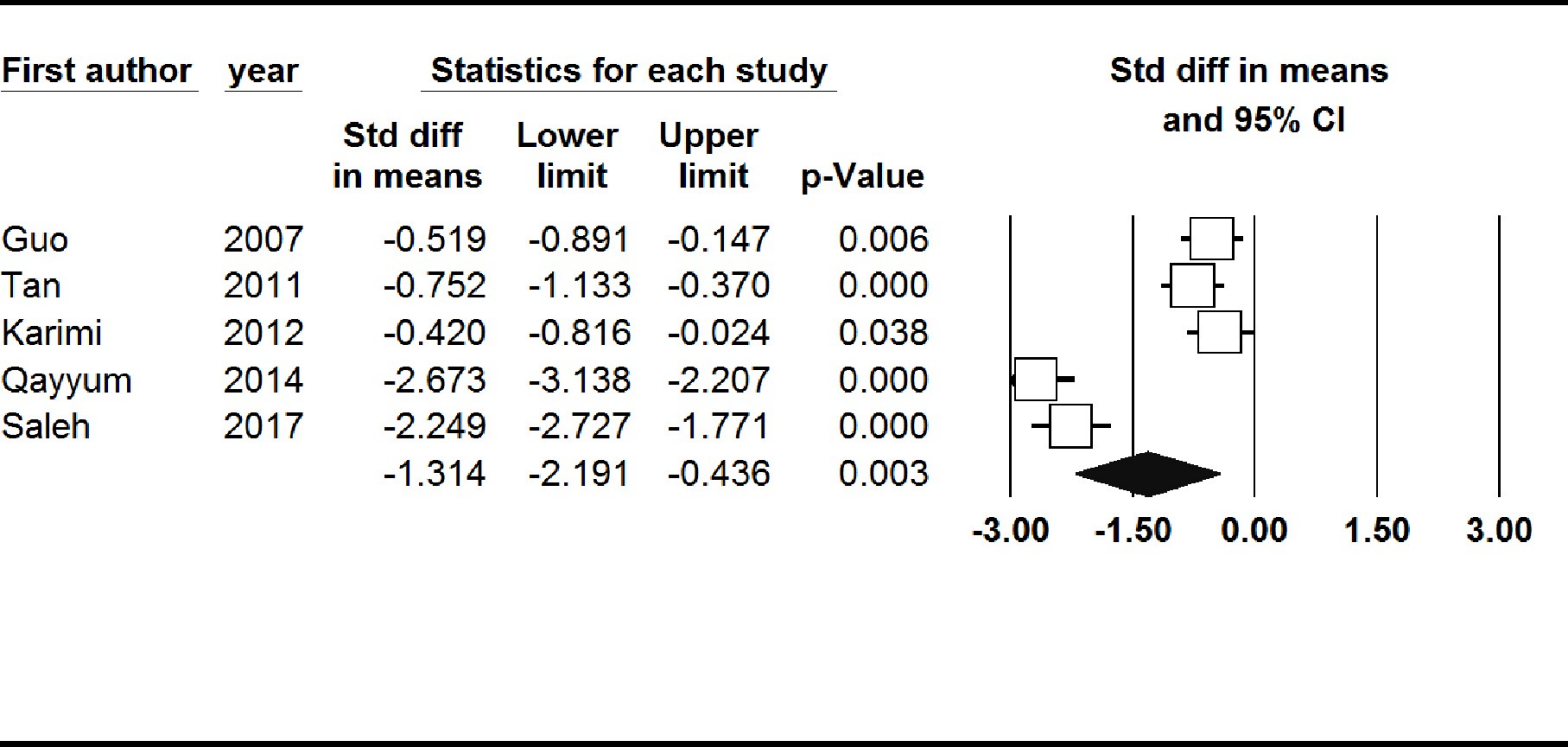
Forest plot of the standardized mean difference for the association between prostate cancer and hair zinc level.

Hair zinc level was significantly lower in patients with prostate cancer (SMD: -1.31, 95% CI: -2.19, -0.44). There was not a significant publication bias based on the Begg’s test (p = 0.46), while the Egger’s test showed a significant publication bias (p = 0.02) (Fig 5).

#### Prostatic fluid or tissue zinc level

**Fig 6:**
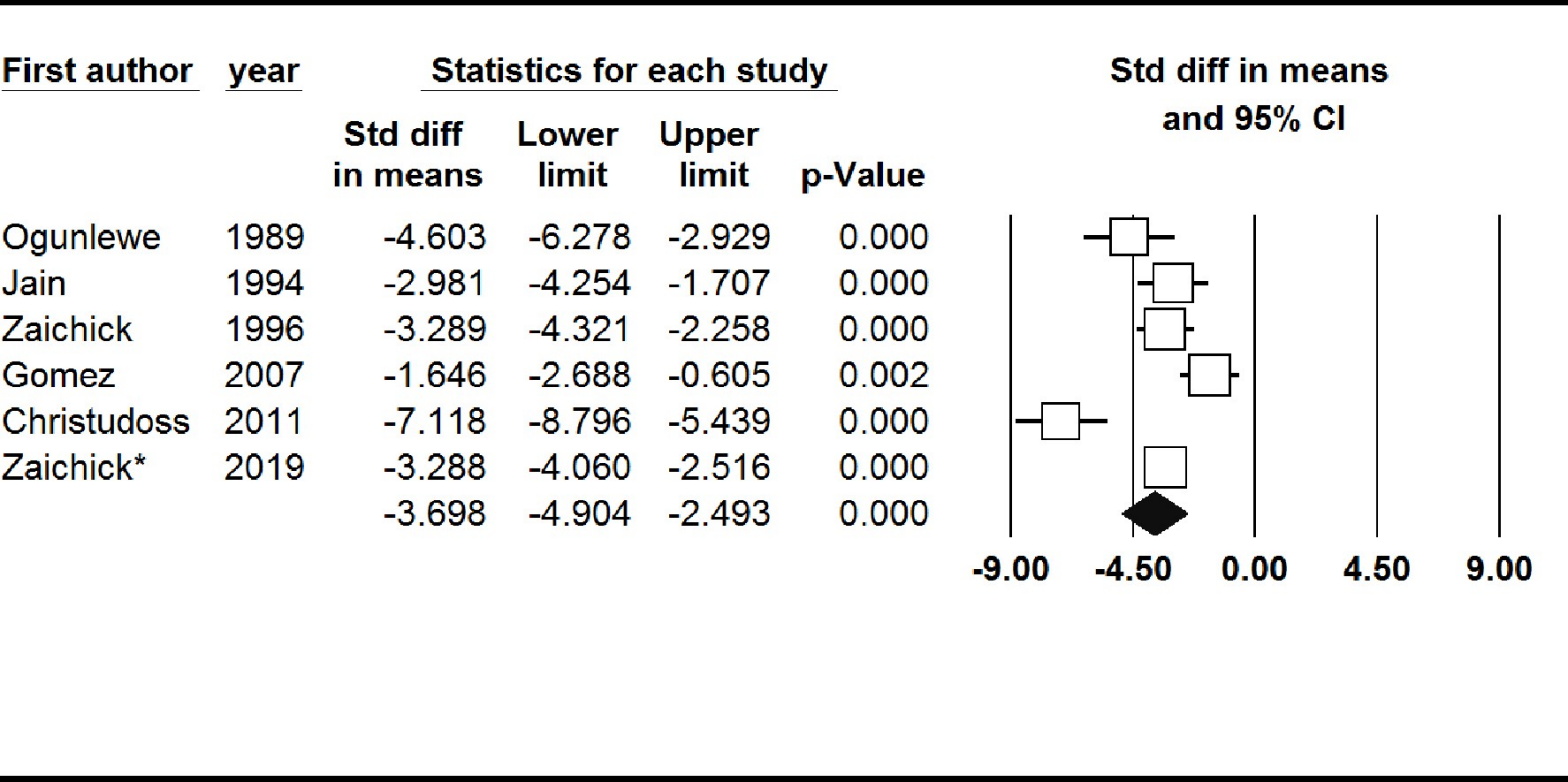
Forest plot of the standardized mean difference for the association between prostate cancer and prostatic fluid or tissue zinc level.

Prostatic fluid or tissue zinc level was significantly lower in patients with prostate cancer (SMD: -3.70, 95% CI: -4.90, -2.49). Also, no significant publication bias was found (Begg’s test: 0.26 and Egger’s test: 0.23) (Fig 6).

### Quality assessment

The mean quality assessment score in case-control studies was 4.61 which ranged from one to eight. Out of 44 case-control studies, one had an overall score of one [94], four had an overall score of two [75, 90, 91, 98], eight had an overall score of three [58, 61, 62, 65, 70, 73, 99, 100], seven had an overall score of four [60, 63, 69, 77, 85, 87, 88], seven had an overall score of five [22, 57, 59, 64, 78, 79, 84], thirteen had an overall score of six [24, 67, 71, 72, 76, 81, 82, 86, 92, 95, 97, 101, 102], three had an overall score of seven [19, 21, 89], and one had an overall score of eight [80]. The selection of control and non-response rates had the lowest score (S3 Table).

The mean risk of bias in cohort studies was eight which ranged from seven to nine. Out of four cohort studies, two had an overall score of seven [26, 74] and two had an overall score of nine [20, 66]. Ascertainment of exposure, outcome, and adequacy of follow-up were the domains with the highest risk of bias (S4 Table).

The mean risk of bias in cross-sectional studies was seven which ranged from six to eight. Out of four cross-sectional studies, two had an overall score of six [93, 96] and two had an overall score of eight [68, 83]. The sample size and non-respondents domains had the highest risk of bias (S5 Table).

## Discussion

Our findings showed that in individuals with prostate cancer, zinc levels of serum, hair, and prostatic fluid or tissue were significantly lower than controls, while no notable differences were found in nail zinc levels and zinc intake.

Numerous studies with a similar design to our study confirmed this finding that patients with prostate cancer have lower serum zinc levels. In this regard, a meta-analysis of ten studies revealed significantly lower serum zinc levels in patients with prostate cancer compared with controls (SMD: −0.94; 95% CI: −1.57, −0.32) [28]. Consistent with our findings, another meta-analysis of 114 studies manifested decreased serum zinc levels in prostate cancer patients (SMD: -1.08; 95% CI: −1.33, −0.82) [25]. It also appears to be similar in other cancers. For instance, a meta-analysis on bladder cancer patients including six studies showed significantly lower serum zinc levels (three studies, SMD: −1.07; 95% CI: −1.49, −0.66) compared with controls [103]. In breast cancer, a meta-analysis of 36 studies with 5747 females showed lower serum zinc levels in cancer patients compared to healthy controls (SMD: -1.20; 95% CI: -1.74, -0.66) [104].

Regarding zinc intake, it showed no significant differences between prostate cancer patients and healthy controls in our study. Another dose-response meta-analysis study showed similar findings (OR: 1.07; 95% CI: 0.98, 1.16) [24]. However, this study was performed on African Americans, and based on studies, black men pose a higher risk of prostate cancer compared to other ethnicities [105]. In addition, zinc intake was self-reported which can explain the differences found between the studies [24]. Yet, this finding is contrary to most of the literature mentioning the anti-tumor effects of zinc supplementation in prostate cancer aside from many other types of cancer [106]. This could bring up a debate on the beneficial dosage of zinc supplements. The controversy on whether excessive dosage would benefit prostate cancer led to studies like a 30-year follow-up study of Zhang et al., which pointed out the risk of lethal prostate cancer with 75 mg/day or more than 15 years of zinc supplementation [26]. However, one of our included studies 19 years before the aforementioned study, followed 46974 health professionals for 14 years and delineated that 100 mg/day of supplemental zinc was a cut-off for the risk of prostate cancer compared to control (RR: 2.29; 95% CI: 1.06, 4.95) [74]. Another systematic review showed that high zinc intake in patients with advanced prostate cancer could be protective in a dose-response manner [107]. As for the risk of advanced prostate disease, it is noteworthy to mention in the study of Gonzalez et al. on 35242 participants, the overall prostate cancer risk was not related to supplemental zinc intake for 10 years (adjusted HR: 0.82; 95% CI: 0.58, 1.14 for >15 mg/day vs. nonuse). However, due to the necessity of the PSA test in detecting the early stage of the disease and since they did not have access to this test, they could not assess the early stages of the disease. Contrary to the findings of zinc supplementation, Gonzalez et al. claimed that the dietary intake of zinc was not associated with the development of prostate cancer [66]. However, this was not the case in other studies done in Hawaii and Italy [64, 72]. As well as zinc supplementation, dietary zinc itself can play a role in preventing prostate cancer. High dietary zinc intake can be associated with reduced mortality in prostate cancer patients after the diagnosis [108]. However, in vivo studies suggest that an optimal dose of zinc intake in the diet is the best amount for protecting against prostate tumors, as both lower and higher than normal levels can lead to carcinogenesis [109]. It is worth mentioning that this association is stronger in advanced prostate cancers [64]. Inconsistent data on the effective dose of zinc can be attributed to the factors affecting the absorption of zinc, such as phytic acid in the vegetables which can inhibit zinc absorption. In addition, when zinc levels are excessively high, its absorption is reduced [66].

However, zinc effects on cancer are tissue-specific and differ amongst various cancers [110]. For example, contrary to prostate cancer, a meta-analysis of 19 studies with 400000 participants showed that higher zinc intake reduced the risk of digestive tract carcinomas particularly colorectal cancers [111].

Zinc has a higher concentration in healthy prostate tissue compared to other tissues. However, in prostate cancer, levels of zinc declined significantly [7]. Accordingly, our results showed significantly lower zinc levels of prostatic fluid and tissue in prostate cancer patients compared to controls (SMD: -3.70, 95% CI: -4.90, -2.49) just like serum zinc levels. It is justifiable because, for the most part, zinc levels in epithelial cells of prostate tissue are highest in a healthy individual, whereas the carcinogenesis process causes depletion in them. This shows the necessity of zinc for the physiologic function of the prostate [28]. One explanation for the lower levels of zinc in prostate cancer tissue would be that cells lose their ability to accumulate zinc when normal cells that produce citrate transform into malignant cells that oxidize citrate [7]. Accumulated zinc blocks the oxidation of citrate in the prostate which is the main component of the prostatic fluid [112]. Zinc has regulatory effects on cell proliferation by modulating DNA synthesis. It has positive effects on DNA maintenance and can prevent DNA damage by affecting DNA polymerase [7]. As well as this, zinc can block the proliferation of prostate cancer cells and stop them at the G2/M checkpoint in the cell cycle. Also, zinc causes up-regulation of genes such as *p21* which helps with the growth inhibition of prostate cancer cells. Zinc plays a protective role by blocking the NF-κB function that would induce the production of angiogenic and metastatic factors like matrix metalloproteinase 9, vascular endothelial growth factor, and interleukin-6. Moreover, zinc reduces hypoxia-inducible factor-1α in prostate cancer cells and can have apoptotic effects by activating caspase-3 and caspase-9, and increasing levels of Bax protein, a pro-apoptotic protein; hence elevating the Bax/Bcl-2 ratio [112]. However, its apoptotic effects are only limited to those cell lines that have not lost their ability to accumulate intracellular zinc. Zinc has anti-oxidant effects in tissues; from lung microsomes to prostate mitochondria, it removes free radicals and prevents production of them [113]. Zinc is enormously involved in the immune system as well and its depletion results in an imbalance of T-cell functions and cytokines release. It is also indicated that zinc transporters play a necessary role in its homeostasis, hence leading to cancer when dysregulated [110].

Sample preparation can have a significant effect on measuring zinc levels. Our included studies involved samples extracted from the serum, prostatic tissue, nails, and hair. Different methods are employed for the sampling process. Variability in sampling procedures might affect the results; however, the studies included have not indicated evidence surrounding the different sample preparations. The most common way of sampling was blood sampling with 5–10 ml of venous blood, mainly from the antecubital region. Then, it was transferred to anti-septic tubes to avoid any probable contamination. The crucial steps in the prevention of contamination with zinc when collecting the samples were taken based on the International Zinc Nutrition Consultative Group [114]. The blood sampling was mostly done in the morning after a complete night of fasting [83]. Having the specimens stored at -20°C, the next step was centrifuging and digesting by adding nitric acid-perchloric acid to them. Then, samples were cooled down and diluted with distilled water [81]. In the end, measuring zinc level was usually done by atomic spectrophotometry, which can be done through the direct method discussed by Smith et al. [115].

For hair samples, the most routine method was collecting a specific length of hair from the scalp by cutting with scissors, for instance, 3.5 cm long. The samples were then stored and purified by washing. After shortening the length, they were mixed with a detergent solution and shaken thoroughly. Digestion was done by adding acids as mentioned above [95]. Nail samples were washed, purified, digested, and finally prepared in the same way [81]. The nail samples could also be digested with a microwave digestion system [80].

A simple protocol was utilized in a study to prepare prostate tissues for Laser Ablation inductively coupled plasma mass spectrometry (ICP-MS) imaging instead of fresh-frozen sampling, formalin fixation, and formalin-fixed paraffin embedding. The two last techniques mentioned result in massive washouts of the target elements which could lead to false negative results [71, 116]. Notably, the elemental data related to hair samples should be normalized in a way that every element be in the range of 0–1 [91].

ICP-MS is a kind of mass spectrometry that allows for the measuring of metals in low concentrations. It has higher sensitivity and accuracy compared to the AAS technique but can interfere with many species when compared to other types of mass spectrometry. For instance, microbes in the glassware, plasma argon, and air leaks through orifices can be named as interfering substances [117]. While the analytical techniques in the measurement of zinc levels vary among the studies included in our review, it is noteworthy that most of them are conducted through the two abovementioned techniques. More precisely, 24 studies used the AAS technique, nine used ICP-MS, and others used various techniques, such as particle-induced X-ray emission, wet acid digestion, and energy-dispersive X-ray fluorescence. Overall, we did not come across any attributable effects of variability in methods used in different studies, meaning that none of the studies mentioned any significant impact of analytical methods on different outcomes.

In other sources of sampling, although hair zinc levels were significantly lower in prostate cancer patients (SMD: -1.31, 95% CI: -2.19, -0.44), there were no significant changes in nail zinc levels. Likewise, another study on prostate carcinoma evaluating trace elements including zinc in hair samples of 18 prostate cancer patients demonstrated significantly lower hair zinc levels in patients [43]. In other tumors like nasopharyngeal cancer, patients had lower zinc levels in hair samples than controls [118]. Similarly, a meta-analysis of seven studies assessing hair zinc levels in breast cancer patients proved lower hair zinc levels in breast cancer patients compared to controls (SMD: −1.99; 95% CI: −3.46, −0.52) [119].

On the whole, the comprehensive results from our study seem to be of pivotal importance, specifically for physicians and policymakers to target zinc for prostate cancer prevention on a global scale. As an example, a systematic review of 23 studies with 1230 patients of mainly head and neck cancer under treatment of zinc concomitant with chemoradiotherapy, speculated that zinc could help reduce mucositis associated with radiotherapy [120]. As many in vivo studies have demonstrated the effective and therapeutic role of zinc administration on prostate cancer models on murine [121–123], perhaps zinc supplements can be considered as a chemo-preventive agent in prostate cancer. Despite this, the issues associated with the bioavailability and toxicity of its routine supplement use should be noticed and need further study [110].

It is an updated systematic review and meta-analysis of the most recent studies that evaluated the effects of zinc on prostate cancer. It has a large sample size and includes studies on zinc levels of different sources as well as zinc supplementation. Nevertheless, we acknowledge that it has several limitations. Although we used random-effect models and stratified analysis due to the high heterogeneity among the included studies, the results should be interpreted with caution because we could not find the probable cause of heterogeneity. Also, we could not access to full texts of ten studies despite contacting the corresponding authors. As well as this, serum zinc levels could fluctuate based on the circadian rhythm [124]; thus, it was not a reliable tool, mainly because research groups had not considered this when assessing serum zinc levels. Studies could miss the diagnosis of prostate cancer due to a lack of symptoms [66]. Also, using more reliable biological samples like toenail zinc levels that can better indicate chronic exposures to zinc, plus repeated testing and collecting of samples, would present higher quality evidence for similar studies [24]. Moreover, we evaluated and compared total zinc concentrations, while free zinc and bound zinc were not assessed. Future primary studies should report other types of zinc status. It should be considered in the interpretation of the results that not only zinc supplementation but also diet itself can influence the zinc levels.

## Conclusions

It appears that serum zinc level is an important factor in the development of prostate cancer. As a result of the inconsistent studies mentioned in the literature, we need more thorough investigations to be able to suggest zinc supplementations as a preventive or therapeutic option for prostate cancer and gain a better insight into this critical question.

## Supporting information

S1 Table Search strategy for PubMed, EMBASE, Web of Science, and the Cochrane Central Register of Controlled Trials (CENTRAL). (DOCX)

S2 Table Characteristics of studies included in the systematic review. (DOCX)

S3 Table Risk of bias assessment for the included case-control studies. (DOCX)

S4 Table Risk of bias assessment for the included cohort studies. (DOCX)

S5 Table Risk of bias assessment for the included cross-sectional studies. (DOCX)
