# Supplementary material for: The association between zinc and prostate cancer development: A systematic review and meta-analysis
Source: PLoS One. 2024 Mar 20;19(3):e0299398. doi: 10.1371/journal.pone.0299398 (PMC10954196; doi:10.1371/journal.pone.0299398)
Supplement: S1 Table — (DOCX) [file pone.0299398.s001.docx]

**S1 Table.** **Search strategy for PubMed, EMBASE, Web of Science, and the Cochrane Central Register of Controlled Trials (CENTRAL).**

| **PubMed** | |
| --- | --- |
| #1 | (((((((((((((Zinc[MeSH Terms]) OR (Zinc Compounds[MeSH Terms])) OR (Zinc Acetate[MeSH Terms])) OR (zinc sulfate[MeSH Terms])) OR ("zinc supplement*"[Title/Abstract])) OR (ZN[Title/Abstract])) OR ("zinc picolinate"[Title/Abstract])) OR ("zinc citrate"[Title/Abstract])) OR ("zinc glycinate"[Title/Abstract])) OR ("zinc monomethionine"[Title/Abstract])) OR (zinc[Title/Abstract])) OR ("Zinc Compound*"[Title/Abstract])) OR ("Zinc Acetate"[Title/Abstract])) OR ("zinc sulfate"[Title/Abstract]) |
| #2 | ((((prostatic neoplasms[MeSH Terms]) OR ("Prostatic Neoplasm*"[Title/Abstract])) OR ("prostate cancer"[Title/Abstract])) OR (prostate malignancy[Title/Abstract])) OR ("prostate neoplasm*"[Title/Abstract]) |
| #3 | ((((((((((((((Zinc[MeSH Terms]) OR (Zinc Compounds[MeSH Terms])) OR (Zinc Acetate[MeSH Terms])) OR (zinc sulfate[MeSH Terms])) OR ("zinc supplement*"[Title/Abstract])) OR (ZN[Title/Abstract])) OR ("zinc picolinate"[Title/Abstract])) OR ("zinc citrate"[Title/Abstract])) OR ("zinc glycinate"[Title/Abstract])) OR ("zinc monomethionine"[Title/Abstract])) OR (zinc[Title/Abstract])) OR ("Zinc Compound*"[Title/Abstract])) OR ("Zinc Acetate"[Title/Abstract])) OR ("zinc sulfate"[Title/Abstract])) AND (((((prostatic neoplasms[MeSH Terms]) OR ("Prostatic Neoplasm*"[Title/Abstract])) OR ("prostate cancer"[Title/Abstract])) OR (prostate malignancy[Title/Abstract])) OR ("prostate neoplasm*"[Title/Abstract])) |
| **EMBASE** | |
| #1 | 'prostate tumor'/exp OR 'prostatic neoplasm*':ti,ab,kw OR 'prostate neoplasm*':ti,ab,kw OR 'prostate cancer':ti,ab,kw OR 'prostate cancer'/exp OR 'prostate malignancy':ti,ab,kw |
| #2 | 'zinc'/exp OR 'zinc derivative':ti,ab,kw OR 'zinc compound*':ti,ab,kw OR 'zinc acetate'/exp OR 'zinc sulfate'/exp OR 'zinc supplement*':ti,ab,kw OR 'zinc picolinate':ti,ab,kw OR 'zinc citrate'/exp OR 'zinc glycinate':ti,ab,kw OR 'zinc monomethionine':ti,ab,kw OR zn:ti,ab,kw |
| #3 | ('prostate tumor'/exp OR 'prostatic neoplasm*':ti,ab,kw OR 'prostate neoplasm*':ti,ab,kw OR 'prostate cancer':ti,ab,kw OR 'prostate cancer'/exp OR 'prostate malignancy':ti,ab,kw) AND ('zinc'/exp OR 'zinc derivative':ti,ab,kw OR 'zinc compound*':ti,ab,kw OR 'zinc acetate'/exp OR 'zinc sulfate'/exp OR 'zinc supplement*':ti,ab,kw OR 'zinc picolinate':ti,ab,kw OR 'zinc citrate'/exp OR 'zinc glycinate':ti,ab,kw OR 'zinc monomethionine':ti,ab,kw OR zn:ti,ab,kw) |
| **Web of Science** | |
| #1 | (((((TS=("prostatic neoplasm*")) OR TS=("prostate cancer")) OR TS=("prostate malignancy")) OR TS=("prostate tumor")) OR TS=("prostate neoplasm*")) OR TS=(prostate) |
| #2 | ((((((((TS=(Zinc)) OR TS=("Zinc Compound*")) OR TS=("Zinc Acetate")) OR TS=("zinc sulfate")) OR TS=("zinc glycinate")) OR TS=("zinc monomethionine")) OR TS=("zinc picolinate")) OR TS=(ZN)) OR TS=("zinc supplement*") |
| #3 | (((((TS=("prostatic neoplasm*")) OR TS=("prostate cancer")) OR TS=("prostate malignancy")) OR TS=("prostate tumor")) OR TS=("prostate neoplasm*")) OR TS=(prostate) AND ((((((((TS=(Zinc)) OR TS=("Zinc Compound*")) OR TS=("Zinc Acetate")) OR TS=("zinc sulfate")) OR TS=("zinc glycinate")) OR TS=("zinc monomethionine")) OR TS=("zinc picolinate")) OR TS=(ZN)) OR TS=("zinc supplement*") |
| **CENTRAL** | |
| #1 | MeSH descriptor: [Zinc] explode all trees |
| #2 | MeSH descriptor: [Zinc Compounds] explode all trees |
| #3 | MeSH descriptor: [Zinc Acetate] explode all trees |
| #4 | MeSH descriptor: [Zinc Sulfate] explode all trees |
| #5 | ("zinc supplement*"):ti,ab,kw OR (ZN):ti,ab,kw OR ("zinc picolinate"):ti,ab,kw OR ("zinc citrate"):ti,ab,kw OR ("zinc glycinate"):ti,ab,kw |
| #6 | ("zinc monomethionine"):ti,ab,kw |
| #7 | #1 OR #2 OR #3 OR #4 OR #5 OR #6 |
| #8 | MeSH descriptor: [Prostatic Neoplasms] explode all trees |
| #9 | ("prostate cancer"):ti,ab,kw OR ("prostate malignancy"):ti,ab,kw OR ("Prostatic Neoplasm*"):ti,ab,kw OR ("prostate neoplasm*"):ti,ab,kw OR ("prostate tumor"):ti,ab,kw |
| #10 | #8 OR #9 |
| #11 | #7 AND #10 |
