# Supplementary material for: The association between zinc and prostate cancer development: A systematic review and meta-analysis
Source: PLoS One. 2024 Mar 20;19(3):e0299398. doi: 10.1371/journal.pone.0299398 (PMC10954196; doi:10.1371/journal.pone.0299398)
Supplement: S2 Table — (DOCX) [file pone.0299398.s002.docx]

**S2 Table. Characteristics of studies included in the systematic review.**

| **First Author** | **Publication year** | **Comorbidities** | **Smoking** | **Alcohol consumption** | **Other risk factors** | **Zinc intake category** |
| --- | --- | --- | --- | --- | --- | --- |
| Yiwen Zhang (1) | 2022 | Diabetes | Case: 5.8%; Control: 5.8% | NA | Family history of prostate cancer: Cases: 12.1%; Controls: 11.6% | intake  Highest: ≥ 75 mg/dl Lowest: Never users; |
| Mehmet Kaba(2) | 2014 | None | None | None | NA | NA |
| KS Adedapo(3) | 2012 | None | None | None | NA | NA |
| Collins Amadi(4) | 2020 | None | None | NA | NA | NA |
| Katarzyna Białkowska(5) | 2018 | None | case: 69%; Control: 67% | NA | None | NA |
| Ayşe Eken(6) | 2016 | NA | NA | NA | NA | NA |
| Yenny Gómez(7) | 2007 | None | NA | NA | None | NA |
| JINGKANG GUO(8) | 2007 | NA | NA | NA | NA | NA |
| Enrique Gutiérrez-González (9) | 2018 | NA | Smoker case: 23%, control: 22%; Former>1y case: 48% and controls: 52% | Yes | NA | NA |
| Martin Igbokwe(10) | 2021 | NA | NA | NA | Occupation | NA |
| Lina Mustafa khedir Abdelmajid(11) | 2022 | None | NA | NA | None | NA |
| Alan R. Kristal(12) | 2010 | Diabetes, high BMI | Current smoker:case:6.6%,control:6.7%, former smoker:case:57.5%,control:58.9% | NA | Family history of prostate cancer | Intake, Diet  control: <9.6 case>17.7  Total  control<13.1 case >31.2 |
| Marion M. Lee(13) | 1998 | BPH, high BMI | Ever smoked cigarettes:case:60.9%, control:62.1% | Ever used alcohol:case:67.2%,control:57% | BPH, prostatitis, socioeconomic factors, high BMI, education, marital status | NA |
| Xiao-Meng Li(14) | 2005 | NA | NA | NA | NA | NA |
| Jue Tao Lim(15) | 2019 | High BMI | Control: 59.6%; case: 46.8% | NA | Family history in first degree relatives | NA |
| Abeer M. Mahmoud(16) | 2016 | NA | Current smoker: case:20%, control: 25%; Former smoker: case:47%, control:40% | Current drinker: case: 37%, control: 52%; Former drinker: 35%, control:23% | High BMI and positive family history | NA |
| Rana Kareem Mohammed(17) | 2015 | NA | NA | NA | NA | NA |
| Augusta Chinyere Nsonwu-Anyanwu(18) | 2022 | None | None | None | None | NA |
| Wasiu Eniola Olooto(19) | 2021 | Obesity | None | None | None | NA |
| Bridget Obiageli Onyema-iloh(20) | 2014 | NA | NA | NA | NA | NA |
| Saleh A. K. Saleh(21) | 2017 | NA | Cancer: 19(32.8%), control healthy: 11(21.2%), BPH: 25(3.1%), total control: 36(31%) | None | None | NA |
| Chao Tan(22) | 2011 | NA | NA | NA | NA | NA |
| H.D. Vlajinac(23) | 1997 | Controls: 11 had asthma, 8 had pneumonia, 8 had a peptic ulcer, 7 had cholecystitis, 6 had angina pectoris, 4 had cirrhosis, 3 had pleuritis and 1 had pancreatitis. | NA | NA | Marital status, education, occupation, place of residence, high BMI, comorbidities | NA |
| Victor C. Wakwe(24) | 2019 | None | NA | NA | None | NA |
| ELIZABETH G. WILLDEN(25) | 1975 | NA | NA | NA | None | NA |
| Hasan Yari(26) | 2015 | NA | NA | NA | NA | NA |
| Vladimir Zaichick(27) | 2019 | NA | NA | NA | NA | NA |
| Michael F. Leitzmann(28) | 2003 | Diabetes | Case: 22%; Control: 20% | NA | Family history of prostate cancer: Cases: 12%; Controls: 12% | NA |
| J.O.Ogunlewe(29) | 1989 | None | NA | NA | NA | NA |
| T.Goel(30) | 2006 | NA | NA | NA | NA | NA |
| Ahmet Aydin (31) | 2006 | None | None | None | NA | NA |
| V.YE.Zaichick(32) | 1996 | NA | NA | NA | NA | NA |
| D.W.West(33) | 1991 | NA | NA | NA | NA | Intake  Highest: > 16 mg  Lowest:<10mg |
| Song-Yi Park(34) | 2013 | NA | NA | NA | NA | NA |
| Alejandro Gonzalez(35) | 2009 | NA | NA | Yes | NA? | NA |
| LAURENCE N. KOLONEL(36) | 1988 | NA | NA | NA | NA | NA |
| A. Feustel(37) | 1986 | NA | NA | NA | NA | NA |
| Golgis Karimi(38) | 2012 | NA | Case: 30 (60%)/ control: 24 (48%) | Case: 24 (48%)/ control: 22 (44%) | NA | NA |
| Silvano Gallus(39) | 2007 | NA | NA | NA | NA | Intake  Highest: >15.65 mg/d  Lowest: ≤9.93 mg/d |
| Elizabeth A. Platz(40) | 2002 | NA | Cases: (current: 7.8% / never: 40.9%), control: (current: 7.0% / never: 43.2%) | NA | NA | NA |
| M. Jain(41) | 1994 | None | NA | NA | NA | NA |
| Alan R. Kristal(42) | 1999 | NA | NA | NA | Increasing age and a family history of prostate cancer | Intake  Highest: ≥7/week  Lowest: Never users; intake |
| M. I. YILMAZ(43) | 2004 | NA | NA | NA | NA | NA |
| Habibe Ozmen(44) | 2006 | None | None | None | NA | NA |
| Swen-Olof Andersson(45) | 1996 | NA | Smokers among cases and controls were 70.7% and 70.8%, respectively | Drinkers of alcohol among cases and controls were 50.3% and 51.6%, respectively | NA | NA |
| Muhammad Abdul Qayyum(46) | 2014 | NA | Case: 41%; Control: 45% | NA | NA | NA |
| Pamela Christudoss(47) | 2011 | NA | NA | NA | NA | NA |
| Saleh A.K. Saleh(48) | 2020 | None | None | None | NA | NA |
| Onyinyechi Bede-Ojimadu(49) | 2023 | Diabetes: Cases 12.2 %, control 28.6% | Never-smokers case 32 (39%), Moderate smokers case 2 (2.4%), Heavy smokers case 48 (58.5%)/Never-smokers control55 (56.1%), Moderate smokers control 213 (13.3%), Heavy smokers control 30 (30.6%) | NA | Family history of prostate cancer: Cases: 17.1%; Controls: 19.4%/ Home proximity to source of metal pollution | NA |
| LOIS D. McBEAN(50) | 1974 | NA | NA | NA | NA | NA |
| A.Feustel(51) | 1989 | NA | NA | NA | NA | NA |
| Yuqing Zhang(52) | 2009 | Obesity | Current: case: 13.4/ control: 28.9 | case 68.6%/ control 66.1% | Family history of prostate cancer: Cases: 10.2%; Controls: 19.4%/ Home proximity to source of metal pollution3.1% | Intake  Control: Never or\1 year use   case: >10 years |

Abbreviations: BMI: body mass index; BPH: benign prostatic hyperplasia; and NA: not available.

**References**

1. Zhang Y, Song M, Mucci LA, Giovannucci EL. Zinc supplement use and risk of aggressive prostate cancer: a 30-year follow-up study. European journal of epidemiology. 2022;37(12):1251-60.

2. Kaba M, Pirincci N, Yuksel MB, Gecit I, Gunes M, Ozveren H, et al. Serum levels of trace elements in patients with prostate cancer. Asian Pacific journal of cancer prevention : APJCP. 2014;15(6):2625-9.

3. Adedapo KS, Arinola OG, Shittu OB, Kareem OI, Okolo CA, Nwobi LN. Diagnostic value of lipids, total antioxidants, and trace metals in benign prostate hyperplasia and prostate cancer. Niger J Clin Pract. 2012;15(3):293-7.

4. Amadi C, Aleme BM. The Prevalence of Zinc Deficiency among Men with and without Prostate Cancer in Port Harcourt, Nigeria. Nutrition and cancer. 2020;72(6):1018-25.

5. Białkowska K, Marciniak W, Muszyńska M, Baszuk P, Gupta S, Jaworska-Bieniek K, et al. Association of zinc level and polymorphism in MMP-7 gene with prostate cancer in Polish population. PloS one. 2018;13(7):e0201065.

6. Eken A, Kaya E, nluEndirlik B, Erdem O, Akay C, Ozgok Y. Evaluation of trace element levels in patients with prostate cancer, benign prostatic hyperplasia and chronic prostatitis. Gulhane Medical Journal. 2016;58:1.

7. Gómez Y, Arocha F, Espinoza F, Fernández D, Vásquez A, Granadillo V. [Zinc levels in prostatic fluid of patients with prostate pathologies]. Invest Clin. 2007;48(3):287-94.

8. Guo J, Deng W, Zhang L, Li C, Wu P, Mao P. Prediction of prostate cancer using hair trace element concentration and support vector machine method. Biol Trace Elem Res. 2007;116(3):257-72.

9. Gutiérrez-González E, Castelló A, Fernández-Navarro P, Castaño-Vinyals G, Llorca J, Salas D, et al. Dietary Zinc and Risk of Prostate Cancer in Spain: MCC-Spain Study. Nutrients. 2018;11(1).

10. Igbokwe M, Salako A, Badmus T, Obiajunwa E, Olasehinde O, Igbokwe C, et al. Tissue Zinc Concentration in Prostate Cancer: Relationship with Prostate Specific Antigen and Gleason Score in a Cohort of Nigerian Men. Asia Pacific Journal of Cancer Biology. 2021;6(2):147-53.

11. khedir Abdelmajid LM, Hessen RIE, Dafalla AM, Hassan MI, Mohammed YA. Serum Zinc and Copper Levels among Patients with Prostatic Cancer Attending National Cancer Institute, Gezira University, Sudan. Sudan Medical Laboratory Journal. 2022;10(2):69-77.

12. Kristal AR, Arnold KB, Neuhouser ML, Goodman P, Platz EA, Albanes D, et al. Diet, supplement use, and prostate cancer risk: results from the prostate cancer prevention trial. Am J Epidemiol. 2010;172(5):566-77.

13. Lee MM, Wang RT, Hsing AW, Gu FL, Wang T, Spitz M. Case-control study of diet and prostate cancer in China. Cancer Causes Control. 1998;9(6):545-52.

14. Li XM, Zhang L, Li J, Li Y, Wang HL, Ji GY, et al. Measurement of serum zinc improves prostate cancer detection efficiency in patients with PSA levels between 4 ng/mL and 10 ng/mL. Asian J Androl. 2005;7(3):323-8.

15. Lim JT, Tan YQ, Valeri L, Lee J, Geok PP, Chia SE, et al. Association between serum heavy metals and prostate cancer risk – A multiple metal analysis. Environment International. 2019;132:105109.

16. Mahmoud AM, Al-Alem U, Dabbous F, Ali MM, Batai K, Shah E, et al. Zinc Intake and Risk of Prostate Cancer: Case-Control Study and Meta-Analysis. PloS one. 2016;11(11):e0165956.

17. Mohammed RK. Evaluation of Copper and Zinc in Sera of Iraqi Male Patients with Prostate Cancer in Baghdad City. Iraqi National Journal Of Chemistry. 2015;15(3).

18. Nsonwu-Anyanwu AC, Icha BE, Nsonwu MC, William MI, Emughupogh KS, Usoro CAO. Assessment of Essential and Non-essential Elements as Risk Evaluation Indices in Men with Prostate Cancer in Calabar South-South Nigeria. Middle East Journal of Cancer. 2022;13(2):285-92.

19. Olooto WE, Oyelekan AA, Adewole OO, Fajobi AO, Adedo AA, Olasimbo O. Serum gonadotropins, cortisol, PSA, and micronutrient levels among men with prostate carcinoma. African Journal of Urology. 2021;27(1).

20. Onyema-Iloh O, Meludu S, Iloh E, Nnodim J, Onyegbule O, Mykembata B. Biochemical changes in some trace elements, antioxidant vitamins and their therapeutic importance in prostate cancer patients. Asian Journal of Medical Sciences. 2014;6.

21. Saleh S, Adly H, Nassir A. Altered Trace Elements Levels in Hair of Prostate Cancer Patients. Journal of Cancer Science & Therapy. 2017;09.

22. Tan C, Chen H. Screening of prostate cancer by analyzing trace elements in hair and chemometrics. Biol Trace Elem Res. 2011;144(1-3):97-108.

23. Vlajinac HD, Marinković JM, Ilić MD, Kocev NI. Diet and prostate cancer: a case-control study. Eur J Cancer. 1997;33(1):101-7.

24. Wakwe VC, Odum EP, Amadi C. The impact of plasma zinc status on the severity of prostate cancer disease. Investigative and clinical urology. 2019;60(3):162-8.

25. WILLDEN EG, Robinson M. Plasma zinc levels in prostatic disease. British Journal of Urology. 1975;47(3):295-9.

26. Yari H, Mohseni M, Vardi R, Alizadeh AM, Mazloomzadeh S. Copper, Lead, Zinc and Cadmium levels in serum of prostate cancer patients by polarography in Iran. J Chem Pharmaceut Res. 2015;7(2):403-8.

27. Zaichick V, Zaichick S. Using prostatic fluid levels of zinc to iron concentration ratio in non-invasive and highly accurate screening for prostate cancer. International Journal of Medical Sciences. 2019;6(11):24-31.

28. Leitzmann MF, Stampfer MJ, Wu K, Colditz GA, Willett WC, Giovannucci EL. Zinc supplement use and risk of prostate cancer. Journal of the National Cancer Institute. 2003;95(13):1004-7.

29. Ogunlewe JO, Osegbe DN. Zinc and cadmium concentrations in indigenous blacks with normal, hypertrophic, and malignant prostate. Cancer. 1989;63(7):1388-92.

30. Goel T, Sankhwar SN. Comparative study of zinc levels in benign and malignant lesions of the prostate. Scandinavian journal of urology and nephrology. 2006;40(2):108-12.

31. Aydin A, Arsova-Sarafinovska Z, Sayal A, Eken A, Erdem O, Erten K, et al. Oxidative stress and antioxidant status in non-metastatic prostate cancer and benign prostatic hyperplasia. Clinical biochemistry. 2006;39(2):176-9.

32. Zaichick VY, Sviridova TV, Zaichick SV. Zinc concentration in human prostatic fluid: normal, chronic prostatitis, adenoma and cancer. International urology and nephrology. 1996;28(5):687-94.

33. West DW, Slattery ML, Robison LM, French TK, Mahoney AW. Adult dietary intake and prostate cancer risk in Utah: a case-control study with special emphasis on aggressive tumors. Cancer Causes Control. 1991;2(2):85-94.

34. Park SY, Wilkens LR, Morris JS, Henderson BE, Kolonel LN. Serum zinc and prostate cancer risk in a nested case-control study: The multiethnic cohort. The Prostate. 2013;73(3):261-6.

35. Gonzalez A, Peters U, Lampe JW, White E. Zinc intake from supplements and diet and prostate cancer. Nutrition and cancer. 2009;61(2):206-15.

36. Kolonel LN, Yoshizawa CN, Hankin JH. Diet and prostatic cancer: a case-control study in Hawaii. Am J Epidemiol. 1988;127(5):999-1012.

37. Feustel A, Wennrich R. Zinc and cadmium plasma and erythrocyte levels in prostatic carcinoma, BPH, urological malignancies, and inflammations. The Prostate. 1986;8(1):75-9.

38. Karimi G, Shahar S, Homayouni N, Rajikan R, Abu Bakar NF, Othman MS. Association between trace element and heavy metal levels in hair and nail with prostate cancer. Asian Pacific journal of cancer prevention : APJCP. 2012;13(9):4249-53.

39. Gallus S, Foschi R, Negri E, Talamini R, Franceschi S, Montella M, et al. Dietary zinc and prostate cancer risk: a case-control study from Italy. European urology. 2007;52(4):1052-6.

40. Platz EA, Helzlsouer KJ, Hoffman SC, Morris JS, Baskett CK, Comstock GW. Prediagnostic toenail cadmium and zinc and subsequent prostate cancer risk. The Prostate. 2002;52(4):288-96.

41. Jain M, Sharma K, Sharma VP. Serum and tissue levels of zinc, copper, magnesium and retinol in prostatic neoplasms. Indian Journal of Clinical Biochemistry. 1994;9(2):106-8.

42. Kristal AR, Stanford JL, Cohen JH, Wicklund K, Patterson RE. Vitamin and mineral supplement use is associated with reduced risk of prostate cancer. Cancer epidemiology, biomarkers & prevention : a publication of the American Association for Cancer Research, cosponsored by the American Society of Preventive Oncology. 1999;8(10):887-92.

43. Yilmaz MI, Saglam K, Sonmez A, Gok DE, Basal S, Kilic S, et al. Antioxidant system activation in prostate cancer. Biol Trace Elem Res. 2004;98(1):13-9.

44. Ozmen H, Erulas FA, Karatas F, Cukurovali A, Yalcin O. Comparison of the concentration of trace metals (Ni, Zn, Co, Cu and Se), Fe, vitamins A, C and E, and lipid peroxidation in patients with prostate cancer. Clinical chemistry and laboratory medicine. 2006;44(2):175-9.

45. Andersson SO, Wolk A, Bergström R, Giovannucci E, Lindgren C, Baron J, et al. Energy, nutrient intake and prostate cancer risk: a population-based case-control study in Sweden. International journal of cancer. 1996;68(6):716-22.

46. Qayyum MA, Shah MH. Comparative study of trace elements in blood, scalp hair and nails of prostate cancer patients in relation to healthy donors. Biol Trace Elem Res. 2014;162(1-3):46-57.

47. Christudoss P, Selvakumar R, Fleming JJ, Gopalakrishnan G. Zinc status of patients with benign prostatic hyperplasia and prostate carcinoma. Indian journal of urology : IJU : journal of the Urological Society of India. 2011;27(1):14-8.

48. Saleh SAK, Adly HM, Abdelkhaliq AA, Nassir AM. Serum Levels of Selenium, Zinc, Copper, Manganese, and Iron in Prostate Cancer Patients. Current urology. 2020;14(1):44-9.

49. Bede-Ojimadu O, Nnamah N, Onuegbu J, Grant-Weaver I, Barraza F, Orakwe J, et al. Cadmium exposure and the risk of prostate cancer among Nigerian men: Effect modification by zinc status. Journal of trace elements in medicine and biology : organ of the Society for Minerals and Trace Elements (GMS). 2023;78:127168.

50. McBean LD, Smith Jr JC, Berne BH, Halsted JA. Serum zinc and alpha2 macroglobulin concentration in myocardial infarction, decubitus ulcer, multiple myeloma, prostatic carcinoma, Down's syndrome and nephrotic syndrome. Clinica Chimica Acta. 1974;50(1):43-51.

51. Feustel A, Wennrich R, Schmidt B. Serum-Zn-levels in prostatic cancer. Urological research. 1989;17(1):41-2.

52. Zhang Y, Coogan P, Palmer JR, Strom BL, Rosenberg L. Vitamin and mineral use and risk of prostate cancer: the case-control surveillance study. Cancer Causes Control. 2009;20(5):691-8.
