# Supplementary material for: The association between zinc and prostate cancer development: A systematic review and meta-analysis
Source: PLoS One. 2024 Mar 20;19(3):e0299398. doi: 10.1371/journal.pone.0299398 (PMC10954196; doi:10.1371/journal.pone.0299398)
Supplement: S3 Table — (DOCX) [file pone.0299398.s003.docx]

**S3 Table.** **Risk of bias assessment for the included case-control studies.**

|  | Year | 1.Adequacy  of case  definition | 2.Representativ-eness  of  cases | 3.Selection  Of  Controls | 4.Definition  of  controls | 5.Comparability  of cases and  controls on the  basis of the  design or  analysis | 6.Ascertainment  of  exposure | 7.Same method of ascertainment  for cases  and controls | 8.Non-Response rate | Overall  Score |
| --- | --- | --- | --- | --- | --- | --- | --- | --- | --- | --- |
| Kaba et al. (1) | 2014 | C | B | A | B | A, B | D | B | C | 3 |
| Adedapo et al. (2) | 2012 | A | A | B | A | A | D | B | C | 4 |
| Amadi et al. (3) | 2020 | A | A | B | A | A, B | D | B | C | 5 |
| Białkowska et al. (4) | 2018 | B | B | A | A | A, B | D | B | C | 4 |
| Eken et al. (5) | 2016 | A | A | B | A | A, B | A | A | C | 7 |
| Gómez et al. (6) | 2007 | B | B | C | B | A,B | D | B | C | 2 |
| Guo et al. (7) | 2007 | B | B | C | B | A, B | D | B | C | 2 |
| Gutiérrez-González et al. (8) | 2018 | B | A | A | A | A, B | C | A | C | 6 |
| khedir Abdelmajid et al. (9) | 2022 | B | B | B | B | A, B | C | A | C | 3 |
| Lee et al. (10) | 1998 | B | A | A | A | A, B | C | A | C | 6 |
| Lim et al. (11) | 2019 | A | A | B | A | A, B | C | A | C | 6 |
| Mahmoud et al. (12) | 2016 | A | A | B | A | A, B | D | A | C | 6 |
| Mohammed et al. (13) | 2015 | B | B | B | A | A | D | A | C | 3 |
| Nsonwu-Anyanwu et al. (14) | 2022 | A | A | B | A | A, B | D | A | C | 6 |
| Olooto et al. (15) | 2021 | A | A | C | A | A | D | A | C | 5 |
| Onyema-iloh et al. (16) | 2014 | C | B | C | B | A | E | B | C | 1 |
| Saleh et al. (17) | 2017 | A | A | C | A | A, B | D | A | C | 6 |
| Vlajinac et al. (18) | 1997 | A | A | B | A | A, B | D | A | C | 6 |
| Willden et al. (19) | 1975 | B | B | C | A | A | D | B | C | 2 |
| Yari et al. (20) | 2015 | A | A | C | A | A, B | D | A | C | 6 |
| Zaichick et al. (21) | 2019 | A | A | B | A | A, B | D | A | C | 6 |
| Ogunlewe et al. (22) | 1989 | A | B | C | B | A, B | A | B | C | 4 |
| Andersson et al. (23) | 1996 | B | A | A | A | A | B | A | C | 5 |
| Kristal et al. (24) | 1999 | A | B | A | B | A | C | B | B | 3 |
| Zaichick et al. (25) | 1996 | A | B | A | B | A | A | B | C | 4 |
| Yilmaz et al. (26) | 2004 | A | B | C | A | A | A | C | C | 4 |
| Ozmen et al. (27) | 2006 | C | B | B | A | A, B | A | A | C | 5 |
| Qayyum et al. (28) | 2014 | A | B | B | A | A, B | A | A | C | 6 |
| Christudoss et al. (29) | 2011 | A | B | B | A | -- | A | B | C | 3 |
| Platz et al. (30) | 2002 | A | A | A | A | A, B | A | A | C | 8 |
| Saleh et al. (31) | 2020 | A | B | C | A | A, B | A | A | C | 6 |
| Bede-Ojimadu (32) | 2023 | A | B | B | A | A | A | A | C | 5 |
| Jain et al. (33) | 1994 | C | B | B | A | A | A | A | C | 4 |
| Feustel et al. (34) | 1989 | A | B | C | A | A | A | B | C | 4 |
| McBean et al. (35) | 1974 | C | B | B | A | -- | A | B | C | 2 |
| Aydin et al. (36) | 2006 | B | B | C | A | A, B | D | B | C | 3 |
| Goel et al. (37) | 2006 | A | B | C | A | A | D | B | C | 3 |
| West et al. (38) | 1991 | A | A | A | B | A, B | C | B | C | 5 |
| Kolonel et al. (39) | 1988 | A | A | A | B | A | B | B | A | 6 |
| Feustel et al. (40) | 1986 | A | B | C | A | - | A | B | C | 3 |
| Karimi et al. (41) | 2012 | A | B | A | A | A, B | A | B | C | 6 |
| Park et al. (42) | 2013 | A | A | A | A | A, B | A | B | C | 7 |
| Gallus et al. (43) | 2007 | A | A | B | - | A | C | A | A | 5 |
| Zhang et al. (44) | 2008 | A | A | A | A | A, B | B | A | C | 7 |

Note: A study can be awarded a maximum of one star for each numbered item within the Selection and Exposure categories. A maximum of two stars can be given for Comparability.

**Selection**

1) Is the case definition adequate?

a) yes, with independent validation *

b) yes, e.g. record linkage or based on self-reports

c) no description

2) Representativeness of the cases

a) consecutive or obviously representative series of cases *

b) potential for selection biases or not stated

3) Selection of Controls

a) community controls *

b) hospital controls

c) no description

4) Definition of Controls

a) no history of disease (endpoint) *

b) no description of source

**Comparability**

1) Comparability of cases and controls on the basis of the design or analysis

a) study controls for _______________ (Select the most important factor.) *

b) study controls for any additional factor * (This criteria could be modified to indicate specific control for a second important factor.)

**Exposure**

1) Ascertainment of exposure

a) secure record (e.g. surgical records) *

b) structured interview where blind to case/control status *

c) interview not blinded to case/control status

d) written self-report or medical record only

e) no description

2) Same method of ascertainment for cases and controls

a) yes *

b) no

3) Non-Response rate

a) same rate for both groups *

b) non respondents described

c) rate different and no designation

**References**

1. Kaba M, Pirincci N, Yuksel MB, Gecit I, Gunes M, Ozveren H, et al. Serum levels of trace elements in patients with prostate cancer. Asian Pacific journal of cancer prevention : APJCP. 2014;15(6):2625-9.

2. Adedapo KS, Arinola OG, Shittu OB, Kareem OI, Okolo CA, Nwobi LN. Diagnostic value of lipids, total antioxidants, and trace metals in benign prostate hyperplasia and prostate cancer. Niger J Clin Pract. 2012;15(3):293-7.

3. Amadi C, Aleme BM. The Prevalence of Zinc Deficiency among Men with and without Prostate Cancer in Port Harcourt, Nigeria. Nutrition and cancer. 2020;72(6):1018-25.

4. Białkowska K, Marciniak W, Muszyńska M, Baszuk P, Gupta S, Jaworska-Bieniek K, et al. Association of zinc level and polymorphism in MMP-7 gene with prostate cancer in Polish population. PloS one. 2018;13(7):e0201065.

5. Eken A, Kaya E, nluEndirlik B, Erdem O, Akay C, Ozgok Y. Evaluation of trace element levels in patients with prostate cancer, benign prostatic hyperplasia and chronic prostatitis. Gulhane Medical Journal. 2016;58:1.

6. Gómez Y, Arocha F, Espinoza F, Fernández D, Vásquez A, Granadillo V. [Zinc levels in prostatic fluid of patients with prostate pathologies]. Invest Clin. 2007;48(3):287-94.

7. Guo J, Deng W, Zhang L, Li C, Wu P, Mao P. Prediction of prostate cancer using hair trace element concentration and support vector machine method. Biol Trace Elem Res. 2007;116(3):257-72.

8. Gutiérrez-González E, Castelló A, Fernández-Navarro P, Castaño-Vinyals G, Llorca J, Salas D, et al. Dietary Zinc and Risk of Prostate Cancer in Spain: MCC-Spain Study. Nutrients. 2018;11(1).

9. khedir Abdelmajid LM, Hessen RIE, Dafalla AM, Hassan MI, Mohammed YA. Serum Zinc and Copper Levels among Patients with Prostatic Cancer Attending National Cancer Institute, Gezira University, Sudan. Sudan Medical Laboratory Journal. 2022;10(2):69-77.

10. Lee MM, Wang RT, Hsing AW, Gu FL, Wang T, Spitz M. Case-control study of diet and prostate cancer in China. Cancer Causes Control. 1998;9(6):545-52.

11. Lim JT, Tan YQ, Valeri L, Lee J, Geok PP, Chia SE, et al. Association between serum heavy metals and prostate cancer risk – A multiple metal analysis. Environment International. 2019;132:105109.

12. Mahmoud AM, Al-Alem U, Dabbous F, Ali MM, Batai K, Shah E, et al. Zinc Intake and Risk of Prostate Cancer: Case-Control Study and Meta-Analysis. PloS one. 2016;11(11):e0165956.

13. Mohammed RK. Evaluation of Copper and Zinc in Sera of Iraqi Male Patients with Prostate Cancer in Baghdad City. Iraqi National Journal Of Chemistry. 2015;15(3).

14. Nsonwu-Anyanwu AC, Icha BE, Nsonwu MC, William MI, Emughupogh KS, Usoro CAO. Assessment of Essential and Non-essential Elements as Risk Evaluation Indices in Men with Prostate Cancer in Calabar South-South Nigeria. Middle East Journal of Cancer. 2022;13(2):285-92.

15. Olooto WE, Oyelekan AA, Adewole OO, Fajobi AO, Adedo AA, Olasimbo O. Serum gonadotropins, cortisol, PSA, and micronutrient levels among men with prostate carcinoma. African Journal of Urology. 2021;27(1).

16. Onyema-Iloh O, Meludu S, Iloh E, Nnodim J, Onyegbule O, Mykembata B. Biochemical changes in some trace elements, antioxidant vitamins and their therapeutic importance in prostate cancer patients. Asian Journal of Medical Sciences. 2014;6.

17. Saleh S, Adly H, Nassir A. Altered Trace Elements Levels in Hair of Prostate Cancer Patients. Journal of Cancer Science & Therapy. 2017;09.

18. Vlajinac HD, Marinković JM, Ilić MD, Kocev NI. Diet and prostate cancer: a case-control study. Eur J Cancer. 1997;33(1):101-7.

19. WILLDEN EG, Robinson M. Plasma zinc levels in prostatic disease. British Journal of Urology. 1975;47(3):295-9.

20. Yari H, Mohseni M, Vardi R, Alizadeh AM, Mazloomzadeh S. Copper, Lead, Zinc and Cadmium levels in serum of prostate cancer patients by polarography in Iran. J Chem Pharmaceut Res. 2015;7(2):403-8.

21. Zaichick V, Zaichick S. Using prostatic fluid levels of zinc to iron concentration ratio in non-invasive and highly accurate screening for prostate cancer. International Journal of Medical Sciences. 2019;6(11):24-31.

22. Ogunlewe JO, Osegbe DN. Zinc and cadmium concentrations in indigenous blacks with normal, hypertrophic, and malignant prostate. Cancer. 1989;63(7):1388-92.

23. Andersson SO, Wolk A, Bergström R, Giovannucci E, Lindgren C, Baron J, et al. Energy, nutrient intake and prostate cancer risk: a population-based case-control study in Sweden. International journal of cancer. 1996;68(6):716-22.

24. Kristal AR, Stanford JL, Cohen JH, Wicklund K, Patterson RE. Vitamin and mineral supplement use is associated with reduced risk of prostate cancer. Cancer epidemiology, biomarkers & prevention : a publication of the American Association for Cancer Research, cosponsored by the American Society of Preventive Oncology. 1999;8(10):887-92.

25. Zaichick VY, Sviridova TV, Zaichick SV. Zinc concentration in human prostatic fluid: normal, chronic prostatitis, adenoma and cancer. International urology and nephrology. 1996;28(5):687-94.

26. Yilmaz MI, Saglam K, Sonmez A, Gok DE, Basal S, Kilic S, et al. Antioxidant system activation in prostate cancer. Biol Trace Elem Res. 2004;98(1):13-9.

27. Ozmen H, Erulas FA, Karatas F, Cukurovali A, Yalcin O. Comparison of the concentration of trace metals (Ni, Zn, Co, Cu and Se), Fe, vitamins A, C and E, and lipid peroxidation in patients with prostate cancer. Clinical chemistry and laboratory medicine. 2006;44(2):175-9.

28. Qayyum MA, Shah MH. Comparative study of trace elements in blood, scalp hair and nails of prostate cancer patients in relation to healthy donors. Biol Trace Elem Res. 2014;162(1-3):46-57.

29. Christudoss P, Selvakumar R, Fleming JJ, Gopalakrishnan G. Zinc status of patients with benign prostatic hyperplasia and prostate carcinoma. Indian journal of urology : IJU : journal of the Urological Society of India. 2011;27(1):14-8.

30. Platz EA, Helzlsouer KJ, Hoffman SC, Morris JS, Baskett CK, Comstock GW. Prediagnostic toenail cadmium and zinc and subsequent prostate cancer risk. The Prostate. 2002;52(4):288-96.

31. Saleh SAK, Adly HM, Abdelkhaliq AA, Nassir AM. Serum Levels of Selenium, Zinc, Copper, Manganese, and Iron in Prostate Cancer Patients. Current urology. 2020;14(1):44-9.

32. Bede-Ojimadu O, Nnamah N, Onuegbu J, Grant-Weaver I, Barraza F, Orakwe J, et al. Cadmium exposure and the risk of prostate cancer among Nigerian men: Effect modification by zinc status. Journal of trace elements in medicine and biology : organ of the Society for Minerals and Trace Elements (GMS). 2023;78:127168.

33. Jain M, Sharma K, Sharma VP. Serum and tissue levels of zinc, copper, magnesium and retinol in prostatic neoplasms. Indian Journal of Clinical Biochemistry. 1994;9(2):106-8.

34. Feustel A, Wennrich R, Schmidt B. Serum-Zn-levels in prostatic cancer. Urological research. 1989;17(1):41-2.

35. McBean LD, Smith Jr JC, Berne BH, Halsted JA. Serum zinc and alpha2 macroglobulin concentration in myocardial infarction, decubitus ulcer, multiple myeloma, prostatic carcinoma, Down's syndrome and nephrotic syndrome. Clinica Chimica Acta. 1974;50(1):43-51.

36. Aydin A, Arsova-Sarafinovska Z, Sayal A, Eken A, Erdem O, Erten K, et al. Oxidative stress and antioxidant status in non-metastatic prostate cancer and benign prostatic hyperplasia. Clinical biochemistry. 2006;39(2):176-9.

37. Goel T, Sankhwar SN. Comparative study of zinc levels in benign and malignant lesions of the prostate. Scandinavian journal of urology and nephrology. 2006;40(2):108-12.

38. West DW, Slattery ML, Robison LM, French TK, Mahoney AW. Adult dietary intake and prostate cancer risk in Utah: a case-control study with special emphasis on aggressive tumors. Cancer Causes Control. 1991;2(2):85-94.

39. Kolonel LN, Yoshizawa CN, Hankin JH. Diet and prostatic cancer: a case-control study in Hawaii. Am J Epidemiol. 1988;127(5):999-1012.

40. Feustel A, Wennrich R. Zinc and cadmium plasma and erythrocyte levels in prostatic carcinoma, BPH, urological malignancies, and inflammations. The Prostate. 1986;8(1):75-9.

41. Karimi G, Shahar S, Homayouni N, Rajikan R, Abu Bakar NF, Othman MS. Association between trace element and heavy metal levels in hair and nail with prostate cancer. Asian Pacific journal of cancer prevention : APJCP. 2012;13(9):4249-53.

42. Park SY, Wilkens LR, Morris JS, Henderson BE, Kolonel LN. Serum zinc and prostate cancer risk in a nested case-control study: The multiethnic cohort. The Prostate. 2013;73(3):261-6.

43. Gallus S, Foschi R, Negri E, Talamini R, Franceschi S, Montella M, et al. Dietary zinc and prostate cancer risk: a case-control study from Italy. European urology. 2007;52(4):1052-6.

44. Zhang Y, Coogan P, Palmer JR, Strom BL, Rosenberg L. Vitamin and mineral use and risk of prostate cancer: the case-control surveillance study. Cancer Causes Control. 2009;20(5):691-8.
