# Supplementary material for: The association between zinc and prostate cancer development: A systematic review and meta-analysis
Source: PLoS One. 2024 Mar 20;19(3):e0299398. doi: 10.1371/journal.pone.0299398 (PMC10954196; doi:10.1371/journal.pone.0299398)
Supplement: S4 Table — (DOCX) [file pone.0299398.s004.docx]

**S4 Table.** **Risk of bias assessment for the included cohort studies.**

|  | Year | 1.Representativeness of the exposed cohort | 2.Selection of the non exposed cohort | 3.Ascertainment of exposure | 4.Demonstration that outcome of interest was not present at start of study | 5.Comparability of cohorts on the basis of the design or analysis | 6.Assessment of outcome | 7.Was follow-up long enough for outcomes to occur | 8.Adequacy of follow up of cohorts | Overall  Score |
| --- | --- | --- | --- | --- | --- | --- | --- | --- | --- | --- |
| Zhang et al. (1) | 2022 | B | A | C | A | A, B | C | A | B | 7 |
| Kristal et al. (2) | 2010 | A | A | B | A | A, B | A | A | A | 9 |
| \| Gonzalez et al. (3) \| 2008 \| \| --- \| --- \| | 2009 | B | A | B | A | A, B | B | A | B | 9 |
| Leitzmann et al. (4) | 2003 | B | A | B | B | A, B | B | A | D | 7 |

Note: A study can be awarded a maximum of one star for each numbered item within the Selection and Outcome categories. A maximum of two stars can be given for Comparability

**Selection**

1) Representativeness of the exposed cohort

a) truly representative of the average _______________ (describe) in the community *

b) somewhat representative of the average ______________ in the community *

c) selected group of users e.g. nurses, volunteers

d) no description of the derivation of the cohort

2) Selection of the non-exposed cohort

a) drawn from the same community as the exposed cohort *

b) drawn from a different source

c) no description of the derivation of the non-exposed cohort

3) Ascertainment of exposure

a) secure record (e.g. surgical records) *

b) structured interview *

c) written self-report

d) no description

4) Demonstration that outcome of interest was not present at start of study

a) yes *

b) no

**Comparability**

1) Comparability of cohorts on the basis of the design or analysis

a) study controls for _____________ (select the most important factor) *

b) study controls for any additional factor * (This criteria could be modified to indicate specific control for a second important factor.)

**Outcome**

1) Assessment of outcome

a) independent blind assessment *

b) record linkage *

c) self-report

d) no description

2) Was follow-up long enough for outcomes to occur

a) yes (select an adequate follow up period for outcome of interest) *

b) no

3) Adequacy of follow up of cohorts

a) complete follow up - all subjects accounted for *

b) subjects lost to follow up unlikely to introduce bias - small number lost - > ____ % (select an adequate %) follow up, or description provided of those lost) *

c) follow up rate < ____% (select an adequate %) and no description of those lost

d) no statement

**References**

1. Zhang Y, Song M, Mucci LA, Giovannucci EL. Zinc supplement use and risk of aggressive prostate cancer: a 30-year follow-up study. European journal of epidemiology. 2022;37(12):1251-60.

2. Kristal AR, Arnold KB, Neuhouser ML, Goodman P, Platz EA, Albanes D, et al. Diet, supplement use, and prostate cancer risk: results from the prostate cancer prevention trial. Am J Epidemiol. 2010;172(5):566-77.

3. Gonzalez A, Peters U, Lampe JW, White E. Zinc intake from supplements and diet and prostate cancer. Nutrition and cancer. 2009;61(2):206-15.

4. Leitzmann MF, Stampfer MJ, Wu K, Colditz GA, Willett WC, Giovannucci EL. Zinc supplement use and risk of prostate cancer. Journal of the National Cancer Institute. 2003;95(13):1004-7.
