# Supplementary material for: The association between zinc and prostate cancer development: A systematic review and meta-analysis
Source: PLoS One. 2024 Mar 20;19(3):e0299398. doi: 10.1371/journal.pone.0299398 (PMC10954196; doi:10.1371/journal.pone.0299398)
Supplement: S5 Table — (DOCX) [file pone.0299398.s005.docx]

**S5 Table. Risk of bias assessment for the included cross-sectional studies.**

|  | Year | 1.Representativeness | 2. Sample size | 3 Non-respondents | 4. Ascertainment of the exposure (risk factor) | 5. Comparability | 6.Assessment of outcome  . | 7. Statistical test: | Overall  Score |
| --- | --- | --- | --- | --- | --- | --- | --- | --- | --- |
| Igbokwe et al. (1) | 2021 | B | A | C | B | A, B | A | A | 8 |
| Li et al. (2) | 2005 | B | B | B | B | A | B | A | 6 |
| Tan et al. (3) | 2011 | B | B | C | B | A | B | A | 6 |
| Wakwe et al. (4) | 2019 | A | A | C | B | A, B | B | A | 8 |

**Selection (maximum five stars)**

1) Representativeness of the sample:

a) Truly representative of the average in the target population* (all subjects or random sampling)

b) Somewhat representative of the average in the target population* (non-random sampling)

c) Selected group of users

d) No description of the sampling strategy

2) Sample size:

a) Justified and satisfactory*

b) Not justified

3) Non-respondents:

a) Comparability between respondents and non-respondents characteristics is established, and the response rate is satisfactory*

b) The response rate is unsatisfactory, or the comparability between respondents and non-respondents is unsatisfactory

c) No description of the response rate or the characteristics of the responders and the non-responders

4) Ascertainment of the exposure (risk factor):

a) Validated measurement tool**

b) Non-validated measurement tool, but the tool is available or described*

c) No description of the measurement tool

**Comparability (maximum two stars)**

1) The subjects in different outcome groups are comparable, based on the study design or analysis. Confounding factors are controlled.

a) The study controls for the most important factor (select one). *

b) The study control for any additional factor. *

**Outcome (maximum three stars)**

1) Assessment of the outcome:

a) Independent blind assessment**

b) Record linkage**

c) Self report*

d) No description

2) Statistical test:

a) The statistical test used to analyze the data is clearly described and appropriate, and the measurement of the association is presented, including confidence intervals and the probability level (p value)*

b) The statistical test is not appropriate, not described or incomplete

**References**

1. Igbokwe M, Salako A, Badmus T, Obiajunwa E, Olasehinde O, Igbokwe C, et al. Tissue Zinc Concentration in Prostate Cancer: Relationship with Prostate Specific Antigen and Gleason Score in a Cohort of Nigerian Men. Asia Pacific Journal of Cancer Biology. 2021;6(2):147-53.

2. Li XM, Zhang L, Li J, Li Y, Wang HL, Ji GY, et al. Measurement of serum zinc improves prostate cancer detection efficiency in patients with PSA levels between 4 ng/mL and 10 ng/mL. Asian J Androl. 2005;7(3):323-8.

3. Tan C, Chen H. Screening of prostate cancer by analyzing trace elements in hair and chemometrics. Biol Trace Elem Res. 2011;144(1-3):97-108.

4. Wakwe VC, Odum EP, Amadi C. The impact of plasma zinc status on the severity of prostate cancer disease. Investigative and clinical urology. 2019;60(3):162-8.
